# Effect of additional treatment with EXenatide in patients with an Acute Myocardial Infarction (EXAMI): study protocol for a randomized controlled trial

**DOI:** 10.1186/1745-6215-12-240

**Published:** 2011-11-08

**Authors:** Martijn Scholte, Leo Timmers, Flip JP Bernink, Robert N Denham, Aernout M Beek, Otto Kamp, Michaela Diamant, Anton JG Horrevoets, Hans WM Niessen, Weena JY Chen, Albert C van Rossum, Niels van Royen, Pieter A Doevendans, Yolande Appelman

**Affiliations:** 1 Department of Cardiology, VU University Medical Center, De Boelelaan 11171081 HV Amsterdam, The Netherlands; 2 Deartment of Cardiology, University Medical Center Utrecht, Room number G02.523, Heidelberglaan 100, 3584 CX Utrecht, The Netherlands; 3 Department of Endocrinology and Diabetes Centre, VU University Medical Centre, PO Box 7057, 1007 MB Amsterdam, The Netherlands; 4 Department of Molecular Cell Biology and Immunology, VU University Medical Center, Van der Boechorststraat 7, 1081 BT Amsterdam, The Netherlands; 5 Department of Pathology, VU University Medical Cente, rPO Box 7057, 1007 MB, Amsterdam, The Netherlands

**Keywords:** exenatide, glucagon-like peptide 1, myocardial infarction, reperfusion injury

## Abstract

**Background:**

Myocardial infarction causes irreversible loss of cardiomyocytes and may lead to loss of ventricular function, morbidity and mortality. Infarct size is a major prognostic factor and reduction of infarct size has therefore been an important objective of strategies to improve outcomes. In experimental studies, glucagon-like peptide 1 and exenatide, a long acting glucagon-like peptide 1 receptor agonist, a novel drug introduced for the treatment of type 2 diabetes, reduced infarct size after myocardial infarction by activating pro-survival pathways and by increasing metabolic efficiency.

**Methods:**

The EXAMI trial is a multi-center, prospective, randomized, placebo controlled trial, designed to evaluate clinical outcome of exenatide infusion on top of standard treatment, in patients with an acute myocardial infarction, successfully treated with primary percutaneous coronary intervention. A total of 108 patients will be randomized to exenatide (5 μg bolus in 30 minutes followed by continuous infusion of 20 μg/24 h for 72 h) or placebo treatment. The primary end point of the study is myocardial infarct size (measured using magnetic resonance imaging with delayed enhancement at 4 months) as a percentage of the area at risk (measured using T2 weighted images at 3-7 days).

**Discussion:**

If the current study demonstrates cardioprotective effects, exenatide may constitute a novel therapeutic option to reduce infarct size and preserve cardiac function in adjunction to reperfusion therapy in patients with acute myocardial infarction.

**Trial registration:**

ClinicalTrials.gov: NCT01254123

## Background

Myocardial infarction (MI) causes loss of cardiomyocytes and may lead to loss of ventricular function, morbidity and mortality. The impairment of left ventricular function may lead to heart failure and death and has a major impact on the quality of life of the patient. Infarct size is a major prognostic factor after MI[1,2]. Reduction of infarct size has therefore been an important objective of strategies to improve clinical outcomes in patients that suffer acute MI.

It was previously demonstrated that early reperfusion of the infarct related coronary artery, usually by primary percutaneous intervention (PCI) limits myocardial necrosis and improves clinical outcome in patients with acute MI[3]. Despite adequate reperfusion, however, most patients still suffer irreversible cardiomyocyte loss. Ironically, part of this loss is induced by the reperfusion itself. Reperfusion induces several biochemical and metabolic changes, which lead to accelerated apoptosis of cardiomyocytes[4]. This phenomenon is referred to as reperfusion injury[5].

Multiple strategies have been investigated to reduce reperfusion injury. One of them is glucose-insulin-potassium (GIK) infusion that has been postulated to possess cytoprotective potential and has been investigated in several clinical trials for cardioprotection in patients with MI. Its efficacy is controversial, with some studies reporting a reduction in mortality in patients with acute MI[6-8], whereas most studies demonstrated no beneficial effect[9-11]. GIK infusion is complicated by volume overload, hypoglycemia and hyperkalemia. A promising alternative is Glucagon-Like Peptide (GLP)-1, a gut incretin hormone, which is released by the gut in response to nutrient intake[12]. It facilitates glucose dependent insulin release and also exerts insulinomimetic actions via the GLP-1 receptor, which is also expressed on cardiomyocytes [13,14]. GLP-1 has been demonstrated to exert cardioprotective effects in animal models by activating anti-apoptotic signaling pathways and by increasing metabolic efficiency[15]. In one small non-randomized clinical study, 21 patients with myocardial infarction and reduced myocardial function were treated with GLP-1 infusion or placebo after successful PCI. GLP-1 treatment resulted in significant improvement of cardiac function[16]. GLP-1 has a short half-life of minutes, being rapidly degraded by dipeptidyl peptidase-4 (DPP-4). Therefore GLP-1 should be administered continuously or administered together with a DPP-4 inhibitor. DPP-4 resistant GLP-1 receptor agonists, such as exendin-4 and its synthetic variant exenatide, have a longer half-life, making them more attractive for clinical application[17]. Exenatide is currently used as blood-glucose lowering-therapy in patients with type 2 diabetes. In a porcine model of ischemia and reperfusion injury, exenatide reduced myocardial apoptosis and oxidative stress. This resulted in reduced infarct size and preserved cardiac performance[18]. Also, in an isolated rat heart model, exendin-4 provided cardioprotection. This effect was abolished by using a GLP-1 receptor antagonist[19]. These data suggest that exenatide exerts a direct cardioprotective effect via the GLP-1 receptor, rather than an indirect effect via increased insulin levels.

The promising clinical results obtained with GLP-1 and with exenatide in the experimental setting constituted the rationale for the initiation of the EXAMI trial. This randomized placebo controlled multi center study will assess the cardioprotective effects of intravenous exenatide infusion on top of standard treatment in patients with ST elevation myocardial infarction undergoing primary PCI.

## Methods

### Overview

The EXAMI trial is a multi-center, prospective, randomized, placebo controlled clinical trial, designed to evaluate the safety and clinical efficacy of exenatide on top of standard treatment, in patients with an acute MI, successfully treated with PCI (http://www.clinicaltrials.gov; unique identifier NCT01254123). The trial is conducted in two Dutch hospitals: the VU University Medical Center in Amsterdam, and the University Medical Center Utrecht. The study was approved by the institutional ethical committee on human research and will be carried out in compliance with the Helsinki Declaration. To be eligible for participation in the trial, patients have to fit the inclusion and exclusion criteria listed in Table 1. Initially, a pilot study will be performed in the VU University Medical Center Amsterdam, in order to assess the safety and feasibility of exenatide infusion in patients with an acute MI. Forty patients will be randomly assigned to treatment with exenatide or placebo on top of standard treatment. An interim analysis will take place after inclusion of these 40 patients. Also patients that initially receive study medication and are subsequently excluded based on exclusion criteria during coronary angiogram or PCI, will be followed for the registration of potential side effects. If significant negative side effects or significant differences are observed in any of the endpoints in the disadvantage of exenatide, the trial will be put on hold. If exenatide infusion is considered safe, the study will be expanded to a total of 108 patients to assess the cardioprotective effects of exenatide following acute MI.

**Table 1:** Inclusion and exclusion criteria

Inclusion criteria	> 18 and < 80 years of age
	First myocardial infarction

	ST elevation of more than one mm in at least 2 separate leads on the electrocardiogram (ECG)

	Delay between onset of sustained chest pain and PCI < 6 hours.

**Exclusion criteria**	Cardiac rhythm is other than normal sinus rhythm.

	Patient in Killip class 3 or 4 of heart failure

	Cardiogenic shock defined as sustained systolic blood pressure ≤ 80 mmHg despite fluid hydration.

	Post cardiac resuscitation

	Need for intra aortic balloon counterpulsation therapy

	The patient is unable to hold his/her breath for up to 20 seconds due to age or concomitant illness

	Former PCI performed

	No re-canalization achieved of the occluded coronary artery

	Culprit *not *in segment 1, 2, 3, 6, 7, 11, 12, 13 of the coronary artery

	No definite culprit

	More than one occluded vessel, or a more than 70% stenosis by visual assessment in a non-culprit vessel.

	TIMI 3 flow in culprit lesion at presentation

	Decreased renal function eGFR < 30 mL/min/1.73 m^2^

	Any contraindication for MRI

	Metal fragments in eye, head, ear, skin or shoulder.

	Swann-Ganz catheter.

	Known pre-existing left ventricular dysfunction measured by any technique (ejection fraction < 45% prior to current admission for myocardial infarction)

	Prior myocardial infarction

	Prior coronary artery bypass grafting

	Moderate to severe cardiac valve disease

	Stroke or transient ischemic attack within the previous 24 hours

	Serious known concomitant disease with a life expectancy of less than one year

	Follow up impossible

	Previous participation in a trial within the previous 30 days

	Known type I Diabetes

### Patient enrolment and randomization

**Figure 1:**
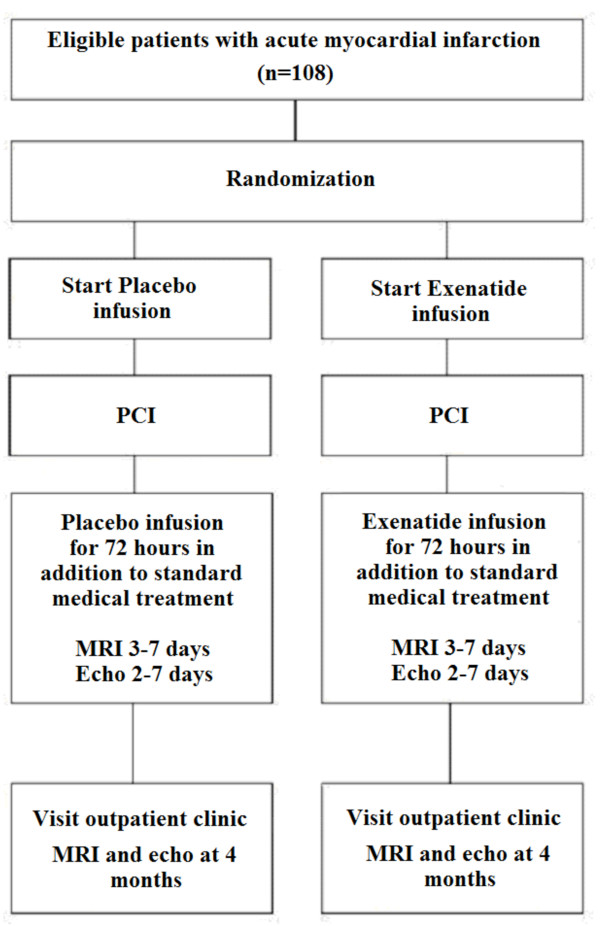
**Flowchart of the study design**. This figure illustrates the study design. A total of 108 patients will be randomized to Exenatide (n = 54) or placebo (n = 54) infusion. The treatment will be initiated just prior to PCI and will be continued for 72 hours. Echocardiography and MRI will be performed during hospital admission (within 2-7 days for echo and within 3-7 days for MRI) and at 4 months follow up.

Patients with an acute ST elevation myocardial infarction to be treated with primary PCI are potential candidates for the study. Patients that are eligible for participation are asked for informed consent. After informed consent, patients are randomized to exenatide or placebo treatment by using sealed envelopes. Only specially assigned nurses will open the envelopes and will start study medication. All patients are also treated with aspirin, heparin and clopidogrel according to the ESC guidelines. After the start of administration of study medication a coronary angiogram (CAG) and if possible primary PCI will be performed. Patients can be excluded during CAG or primary PCI based on the criteria listed in table 1. The final CAG will be performed for measurement of Thrombolysis in Myocardial Infarction (TIMI) frame count and TIMI blush grade. During follow up, all patients will be treated with aspirin, clopidogrel, aggressive lipid-lowering treatment, ACE inhibitor or ARB, and beta-blocker, unless contraindicated. The use of GPIIb/IIIa inhibitors will be left to the discretion of the operator. The flowchart of the study design is shown in Figure 1.

### Exenatide treatment protocol

Byetta injection pens (Amylin Pharmaceuticals, San Diego, CA, USA) containing 1, 2 ml exenatide (concentration 0, 25 mg/ml; 60 doses of 5 μg) will be obtained. Three doses (15 μg) will be diluted in 49 ml saline and 1 ml Human Serum Albumin (Cealb 200 g/L, 10 ml) to obtain an exenatide concentration of 0.30 μg/ml. Infusion will start immediately after informed consent, just prior to PCI. During the first 30 minutes it will be infused intravenously at 33.33 ml/h (5 μg bolus in 30 minutes), followed by intravenous infusion at 2.78 ml/h (20 μg/24 h) for 72 hours. New solutions will be made every 8 hours. Based on experiences with healthy subjects, this is the maximum well-tolerated intravenous dose.

Nausea is a side effect of exenatide as it slows gastric emptying. It occurs in 40-50% of the patients, but is usually mild, and severe nausea is reported in 3.5% of the patients[20]. Nausea will be treated with metoclopramide 20 mg TID. If the nausea does not decrease in response to metoclopramide, the dose of study medication will be lowered to 50%. When serious side effects remain during infusion, study medication will be stopped.

### Blood analysis and blood glucose level monitoring

**Figure 2:**
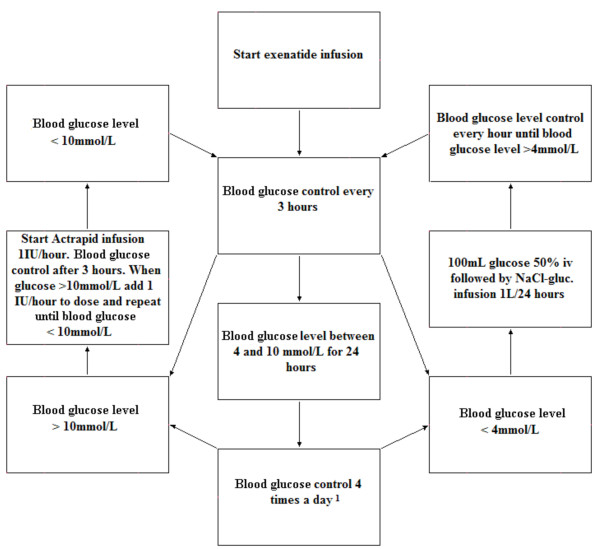
**Blood glucose monitoring and control**. The blood glucose levels are targeted between 4-10 mmol/L. Hypoglycemia will be treated with intravenous glucose infusion and hyperglycemia with intravenous insulin infusion as indicated in the flowchart.

During hospital admission and follow up visits blood analysis will be performed. An overview of blood analysis during the study is listed in table 2. Blood glucose levels will be monitored every 3 hours during the first 24 hours of study medication treatment, or when hypoglycemia is clinically suspected (Figure 2). When no hypoglycemic (glucose level < 4 mmol/L) or hyperglycemic (glucose level > 10 mmol/L) episodes occur during the first 24 hours of study medication treatment, blood glucose levels will be monitored 4 times a day until the end of admission. In case of hypoglycemia patients will be treated with 100 ml glucose 50% infusion and subsequently with glucose 5% 1 L/24 hours. During a hypoglycemic period blood glucose levels will be assessed every hour until blood glucose levels are > 4 mmol/L. During hypoglycemic periods study medication will be continued.

**Table 2:** Blood analysis during the study

Laboratory measurements on admission:	ESR; CRP; hemoglobin; hematocrit; platelet count; leucocytes; leukocyte differential; sodium; potassium; creatinin; urea; AST; ALT; LDH; bilirubin; CK; CK-MB; troponin; total cholesterol; LDL cholesterol; HDL cholesterol; plasma glucose; HbA1c; NT-proBNP; albumin; homocysteine; Lipoproteine(a); Insulin; C-peptide; Free Fatty Acids; APTT; INR;
**Routine laboratory measurements (every morning at 9:00)**	CRP, hemoglobin; hematocrit; platelet count; leucocytes; sodium; potassium; creatinin; exenatide serum level (during duration of study medication), insulin, C-peptide.

**Other laboratory measurements**	On the fourth day after admission a triglyceride level and a blood glucose level will be obtained in the fasting state. Exenatide serum levels, insulin and C-peptide will be measured 4 h after initiation of treatment.

**Laboratory measurements at follow up visit at 1 month and 4 months**	ESR; CRP; hemoglobin; hematocrit; platelet count; leucocytes; sodium; potassium; creatinin; AST; ALT; LDH; total cholesterol; LDL cholesterol; HDL cholesterol; blood glucose level; HbA1c; NT-proBNP; albumine; Lipoproteine (a)

Patients who are hyperglycemic at presentation will be treated with intravenous actrapid as long as necessary to maintain blood glucose levels between 4 and 8 mmol/L. Actrapid will be started at a dose of 1 unit/hour and the dose will be adjusted according to blood glucose levels that will be checked every three hours.

Mild to moderate hypoglycemia is expected in approximately 5% of the patients, severe hypoglycemia is not expected[20].

### Magnetic Resonance Imaging (MRI)

Three to 7 days and 4 months after primary PCI a cardiac MRI with delayed contrast enhancement (DCE) will be performed to assess left ventricular function and infarction size. The first MRI is performed to visualize myocardial edema, i.e. the area at risk. The second MRI is preformed to measure myocardial fibrosis, i.e. infarct area. Doing so, infarct size can be measured as a percentage of the area at risk. Patients are studied on a clinical 1, 5 Tesla scanner with a cardiac phased array receiver surface coil placed on the thorax.

ECG-gated cine MR images are obtained during repeated breath-holds in the 3 standard long axis views (4-, 3- and 2-chamber view). Additional short axis slices are acquired covering the entire left ventricle to examine regional and global left ventricular function. T2-weighted imaging will be acquired in long and short axis views and will be used to estimate the area at risk[21-23]. During intravenous injection of 0, 10 mmol/kg gadolinium-diethyltriaminepenta-acetic acid, first-pass perfusion imaging is performed. Delayed contrast enhanced images are acquired 10 minutes post-contrast to identify the location and extent of myocardial infarction.

All images are stored on a database where it will be forwarded for later blinded analysis. The MRI data are analyzed using a software package (Mass, Medis, Leiden, the Netherlands). On the short axis cine slices, the endocardial and epicardial borders are outlined manually in end-diastolic and end-systolic images, excluding trabeculae and papillary muscles. Assessment of global left ventricular function is obtained by calculating left ventricular volumes, mass, and ejection fraction using the summation of slice method multiplied by slice distance. For analysis of segmental myocardial function, each short axis slice is divided in 12 equiangular segments, starting at the posterior septal insertion of the right ventricle. Segmental wall thickening is expressed in absolute values (end-diastolic wall thickness subtracted from end-systolic wall thickness [in millimeters]) and relative values (absolute wall thickening divided by end-diastolic wall thickness [%]). Areas of hyperenhancement are outlined, including central dark zones of microvascular obstruction, allowing the calculation of total infarct size by summation of all slice volumes of hyperenhancement.

### Echocardiography

Two to 7 days and 4 months after primary PCI, transthoracic echocardiography will be performed to measure global and regional cardiac function, using the iE33 ultrasound system (Philips Medical Systems, Andover, MA, USA) equipped with a S5-2 phased array (1 to 5 MHz broadband) transducer (Philips Medical Systems, Andover, MA, USA). A systematic imaging protocol will be performed, consisting of parasternal long-axis, short-axis and 4-, 2-, and 3-chamber apical views, closely following current guidelines. Global function of the left ventricle will be assessed with biplane Simpson's method. Global right ventricular function will be measured with the functional parameter tricuspid annular plane systolic excursion (TAPSE). Mitral, tricuspid, pulmonary and aortic valve will be assessed with color Doppler, measuring filling times, E- and A-top of the mitral valve and valvular function. In addition, the diastolic left ventricular function will be assessed with coded tissue Doppler imaging measuring peak tissue velocity during early diastole (E') at the basal left ventricular septum and lateral wall. Finally, using the same ultrasound system equipped with a X3-1 matrix-array transducer (Philips Medical Systems, Andover, MA, USA) a complete real-time 3-dimensional echo dataset will be obtained for blinded off-line analysis of global and regional left ventricular function.

### Follow up

All patients visit our outpatient clinic at 6 weeks and at 4 months after primary PCI. The visits consist of clinical evaluation, blood analysis, and 12-lead electrocardiogram. Major cardiovascular events (death, myocardial re-infarction, coronary artery bypass grafting, repeat PCI) will be documented. Furthermore, cardiac arrhythmias, heart failure, repeat coronary angiography, stroke, and hospital admission) are documented. At 4 months a second MRI and echocardiography will be performed to assess cardiac function and infarct size.

### End points

The primary endpoint of the study is myocardial infarct size as a percentage of the area at risk (i.e. the perfusion territory of the infarct related coronary artery) as measured by MRI. The area at risk is measured 3-7 days after primary PCI and myocardial infarct size is measured 4 months after primary PCI. All secondary endpoints are indicated in table 3.

**Table 3:** Endpoints

Primary endpoint	Infarct size measured as the final infarct size on delayed contrast enhancement (DCE) MRI at 4 months as a percentage of the area at risk on T2 weighted MRI at 3-7 days.
**Secondary endpoints**	Myocardial salvage index and final infarct size measured by MRI

	Regional cardiac function based on echocardiographic and MRI segmental analysis at 2-7 days and at 4 months (for echo) and at 3-7 days and at 4 months (for MRI).

	Global cardiac functional parameters measured by echocardiography and MRI

	Myocardial infarct size as measured by serum CK-MB release during the first 72 hours after PCI.

	Microvascular obstruction measured by DCE MRI 3-7 days after PCI.

	Blood pressure and heart rate at 1 day, 7 days and at 4 months

	The occurrence within 4 months of a Major Adverse Cardiac Event (MACE) defined as cardiac death, myocardial infarction, coronary bypass grafting, or a repeat PCI.

	Repeat PCI in the infarct related artery during 4 months follow up.

	Plasma glucose levels during the first 72 hours.

	Side effects of exenatide

	Angiographic parameters as Thrombolysis in Myocardial Infarction (TIMI) frame count and TIMI blush grade

### Sample Size

Myocardial infarct size as a percentage of the area at risk will be the primary endpoint of the study. With a power of 0.9 and an alpha error of 0.05, and with an anticipated 15% reduction in myocardial infarct size in the treatment arm considered clinically relevant, 48 patients per treatment arm have to be enrolled. To allow for dropout of 10% (based on previous experience), a total of 108 patients will be enrolled.

### Statistical Analysis

The study results will be evaluated based on intention to treat analysis. Depending on the distribution of continuous outcomes, independent samples t-test or Mann-Whitney U test will be used as appropriate. Binary end points will be compared using Fisher exact probability test. For survival and clinical events, Kaplan Meijer curves will be displayed and comparison of the curves will be done using log rank testing based on a proportional hazards model.

## Conclusion and implications

The EXAMI study, as outlined above, is a randomized clinical trial to investigate the cardioprotective properties of exenatide in patients with acute MI undergoing primary PCI, by means of measuring final infarct size compared to area at risk as measured with MRI. If reduction in infarct size is accomplished, exenatide may constitute a valuable therapeutic auxiliary for reperfusion therapy in patients with acute MI.

### Trial status

Currently including patients

## Competing interests

The authors declare that they have no competing interests.

## Authors' contributions

MS made contributions to conception and design and of the trial, data acquisition and has been involved in drafting the manuscript. LT contributed to conception and design of the trial and drafted the manuscript. FB, RD and WC contributed to data acquisition and critically revised the manuscript for important intellectual content. AB contributed to trial design, designed the MRI protocols and critically revised the manuscript for important intellectual content. OK contributed to trial design, designed the echocardiography protocols and critically revised the manuscript for important intellectual content. MD, AH, HN, AR, NR, PD and YA contributed to conception and design, and critically revised the manuscript for important intellectual content. All authors have given final approval of the version of the manuscript to be published.
